# *In situ* study of nucleation and aggregation phases for nanoparticles grown by laser-driven methods

**DOI:** 10.1038/srep41372

**Published:** 2017-02-14

**Authors:** M. Barberio, P. Antici

**Affiliations:** 1 INRS-EMT, 1650 Boul. Lionel Boulet, Varennes, J3X1S2, Canada; 2 ELI-ALPS, PATHz Division, Tsiza Lajos krt, 85-87, Szeged, Hungary; 3 INFN/University of Rome «La Sapienza» P.zzale A. Moro 5, 00185, Rome, Italy

## Abstract

In the last decades, nanomaterials and nanotechnologies have become fundamental and irreplaceable in many fields of science and technology. When used in applications, their properties depend on many factors such as size, shape, internal structure and composition. For this, exact knowledge of their structural features is essential when developing fabrication technologies and searching for new types of nanostructures or nanoparticles with specific properties. For the latter, the knowledge of the precise temporal evolution of the growth processes is fundamental when it comes to industrial production and applications. Here we present a method to control, with very high precision, the starting of the aggregation phase during the Laser Ablation in solution growth process. This is obtained by monitoring the optical absorption of the colloidal solution. We apply this control method on the most popular metallic nanoparticle materials (Ag, Al, Co, and Ti) and verify the technique using morphological analysis conducted by AFM and SEM microscopy. The experimental results are explained in terms of Mie extinction theory and Thermal Model for Laser Ablation.

In recent times, metal and semimetal nanoparticles (NP) have attracted great attention by the scientific community due to their unique properties. These properties depend on the size, shape and chemical environment^1^,^2^, and differ from the corresponding bulk material. Among most popular nanoparticles, silver and gold nanoparticles, and due to their optical and electrical properties, are highly employed for applications in various scientific areas such as catalysis, optics, nanotwizers, nanoelectronics etc^3^. In general, noble metal nanoparticles have many applications in the field of life sciences, particularly when used as antibacterial element, in the nanofabrication, and in the development of superhydrophobic surfaces for the manipulation and analysis of diluted biological solutions.

Different preparation methods exist to growth nanoparticles, nanocrystals and quantum dots, where each method is more adapted to generate one single category of nanoparticle with different size and shape. The growth mechanisms of these NP are often difficult to understand in detail since little changes in the experimental setup can considerably influence the properties of the obtained materials. The growth techniques that are most commonly used are Chemical Reduction and Laser Ablation in vacuum, which can be performed in controlled gaseous atmosphere or in liquid (LASiS). The main problem of these chemical methods is the purification of the surface of the obtained NP from the residual ions, this latter being able to affect the particle properties. Instead, Laser Ablation (LA) techniques lack of providing a highly controlled particle size and of allowing purification from the surfactant used as solvent. In particular, the preparation of nanoparticles using LASiS has the advantage of not needing vacuum, and as such enables achieving nanoparticles with higher purity produced in diverse solutions (it allows choosing the solution most suitable for their application). As a result, many parameters influence the ablation process and consequently the growth of nanoparticles. In the last years, many theoretical and experimental studies have been made to better understand the influences of the different parameters on the particle growth^4^,^5^. In the LASiS, the NPs are obtained by irradiation of a pure metal or semiconductor with a pulsed laser (YAG or Kr-F). Laser and material parameters, such as the bulk target, solvent and solutes, system temperature and pressure, laser wavelength, duration of irradiation, strongly affect the shape and dimensions of the produced nanoparticles^4^. Changes in one or several of these parameters can result in the production of very different NPs in both sizes and dimensions. In this scenario, the ability to precisely control the characteristics of NPs by choosing appropriate laser and materials parameters is strategic and requires a detailed understanding of the basic LASiS process^4^.

The main stages in the LASiS process can be resumed as follows: the process starts with the absorption of the laser pulse energy by the bulk target, after this, a plasma plume containing the ablated material expands into the surrounding liquid, accompanied by the emission of a shockwave. During the expansion, the plasma plume cools down and releases energy to the liquid solution. This phenomenon generates a cavitation bubble inside the bulk target, bubble which expands in the liquid and then collapses in a time scale in the order of hundreds of microseconds, by emission of a second shockwave. The nucleation of the nanomaterials from the desorbed atoms is estimated to occur in a timeframe ranging from 10^−6^ and 10^−4^ s after the impact of the laser bunch on the surface (the laser has a pulse duration of the order of ns). As a result, all the mechanical and geometrical properties (i.e., shape, dimensions, crystallographic, density, etc.) of the produced NPs are strictly dependent on the laser parameters while the evolution time of the system (plasma plume system) is strictly related to the pulse duration of the irradiating laser.

These first phases of rapid nanoparticle nucleation are generally followed by nanocrystal aggregation with formation of amorphous nanoparticles, nanorods or nanowires. An initial induction period, leading to the NP size increase, corresponds to the classical nucleation and growth regime (regime C), which produces primary nanocrystals in a diameter range of a few nm (such as the process described above). This regime may be followed by a second induction and growth period associated with aggregative nucleation and growth (region A). For long aggregation times, nanoparticles aggregate, forming greater amorphous clusters (region OR). Significant efforts have been necessary in defining a simple and repeatable synthesis process, allowing a perfect control of the produced nanocrystals.

Large-scale use of nanomaterials, such as found e.g. in industrial applications, requires a controllable and repeatable synthesis, with a process lasting from a few msec to seconds. This calls for a standardized and controlled growth protocol.

In order to define a controlled synthesis process driven by the optical absorption analysis of a colloidal solution, we analyze the nucleation and aggregation phases of 4 different metal nanoparticles (Ag, Al, Co, and Ti). For each nanoparticle production method, we standardize a protocol for the laser irradiation, identifying the beginning of the aggregation phases, the surface temperature, and the amount of the ablated material from the target surface.

## Materials and Methods

### Experiments

Metal nanoparticles (MNPs) were grown by the Laser Ablation in solution (LASiS) method. A metal plate, with nominal purity of 99.99% and dimensions of about 1 cm x 1 cm, is placed on the bottom of a vessel cuvette containing an aqueous solution of acetone (90%). We chose as solvent acetone (oxygen-free ambient) in order to prevent the oxidation of the nanoparticles during the synthesis process. The plate is ablated with the first harmonic (1064 nm) of a pulsed YAG:LASER (7100 series of QUANTA SYSTEM). The laser spot size on the target surface is of about 1 cm^2^. The laser fluence is varied from 50 mJ/pulse to 500 mJ/pulse to investigate the kinetics of the irradiation.

The growth process is monitored by measurements of solution optical absorption. Analysis of the optical absorption and related extinction cross section is a useful technique to have rapidly information on the size of a nanoparticle. The extinction cross section of a particle, as described in the next section, is strictly dependent on its dimensions and therefore the position of the plasmonic peak changes strongly with the particle diameter.

**Figure 1:**
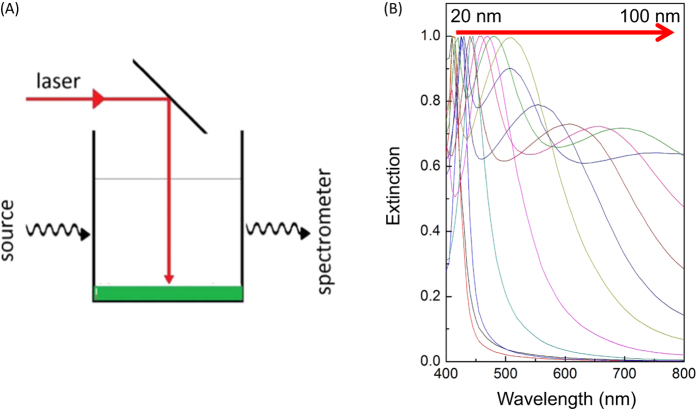
Experimental setup and theoretical database generated from our algorithm. (**A**) Sketch of the Laser Ablation experimental setup, (**B**) representative set of Mie spectra for Ag NPs, the plot shows curves for particles with radius ranging from 10 to 100 nm (from left to right).

The laser source used for the LASiS reaches the target from the top surface of the cuvette while the white lamp (Energetiq LDLS, Laser Driven Light Source) and UV-VIS spectrometer (TRIAX 320 from Horiba-Jobyn–Yvon) used for the optical absorption measurement are placed normally to the laser beam, illuminating the entire liquid region occupied by the plasma plume. The white light source and the optical transmission are transferred between sample and equipment with a series of optical lenses and fibers. All the software for the optical absorption analysis has been prepared and tested in previous experiments by the authors^6^,^7^. During the LASiS, a series of droplets of the colloidal solution are deposed, at different irradiation times, onto a silicon dioxide substrate for being used by *ex-situ* diagnostics (i.e., AFM, TEM, SEM, microscopy, and EDX analysis). A sketch of the experimental setup is shown in Fig. 1A. Morphological information (i.e. particle shape, dimensions, density and roughness) of the nanomaterials is obtained by AFM, TEM, and SEM microscopies.

AFM images are obtained by an ICON AFM microscope (Bruker) working in the tapping mode. Each image is taken with a resolution of 512 × 512 and 1024 × 1024 pixels and a frequency of 1 Hz. Dimensions of the MNPs are analyzed conducting a statistical analysis on many nanoparticles collected in several AFM images. For each sample, we scan several areas (about 50) in a window of 500 nm x 500 nm (resolution of 1024 × 1024 pixels). The images are, then, elaborated by the Nanoscope software (1.40 version from Bruker) to obtain the 3D structure and to measure the NP dimensions. The radius of each nanoparticle is evaluated assuming that a spherical nanoparticles rests onto a SiO_2_ surface and warps during the morphological analysis, forming a spherical cap–like structure. The particle’s warp is produced due to the interaction with the AFM tip which works with an average force of about 1 μN and, thus, exerting a pressure on the NP surface of the order of GPa^8^. We evaluate the volume of each NP by a Bearing analysis, and the radius assuming that the volume of the spherical particle is conserved despite the distortion process.

Chemical composition of the NPs is performed by means of Energy Dispersive X-ray (EDX) spectroscopy taken simultaneously with the image acquisition under SEM conditions and X-ray Diffraction (XRD) spectroscopy.

### Theoretical analysis

The optical absorption of the colloidal solution is monitored during the entire irradiation process. We take the transmitted spectra in all the UV-VIS range (300–800 nm) at regular intervals of 0.5 s during the irradiation process (the acquisition time for each spectrum was fixed at 5 ms), the extinction cross-section (which allow retrieving the optical absorption) is then evaluated using the standard equations:


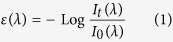


where I_t_ and I_0_ are, respectively, the transmitted and source intensity at each wavelength. The obtained extinction spectra are evaluated following the exact Mie Theory^9^,^10^. When a spherical metallic nanoparticle is irradiated by light, the oscillating electric field causes a coherent oscillation of the conduction electrons. The irradiation of light displaces the electron cloud with respect to its regular nuclei distribution while the Coulomb attraction of the nuclei tends to restore the electron cloud in its original position. This results in an electron cloud oscillation, which causes an extinction of the incident light and the coloration of the solution (the size and shape of the NPs determine the resonance plasmonic frequency). The solution of Maxwell’s equations for the system in spherical coordinates using the multi poles expansion of the electric and magnetic fields and accounting for the discontinuity of the dielectric constant between the spherical particle of radius *R* and the surrounding medium gives the “Mie extinction cross section”:


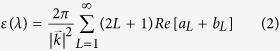


where


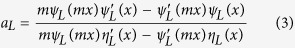



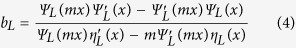



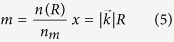


and *L* is the multipolar order (set to be 3), *k* the incident photon wave vector, ψ_*L*_ and η_L_: spherical Riccati-Bessel functions, *n*_m_ the real refraction index of the solvent, and finally *n*(*R*) the complex refraction index of the sphere. We implement the equations from 2 to 5 in a customized Mathematica Algorithm and evaluate a table of extinction cross sections for each analyzed metal for determining the spherical particle size considering a radius ranging from 0.1 to 100 nm (at regular intervals of 0.1 nm). A selected significative subset of the theoretical extinction cross sections evaluated for Ag NPs and using our algorithm is shown in Fig. 1B.

Experimental absorption curves are complex curves due to the scattering of particles with different dimensions, shapes and also due to different coating of the radicals. The NPs’ size distribution can be well described by a Log-Normal function^11^:


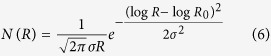


The set of experimental absorption curves is, then, fitted with the Mie extinction cross section in eq. 3 convoluted with the Log-Normal distribution function in eq. 6. The fitting algorithm, implemented with customized Mathematica scripts, first selects the correct cross section from a database (thus indicating the best value for the NP radius *R*_*0*_) and then evaluates the convolution with a LogNorm distribution for the values of the parameter R ranging from 0.1 to 100 nm and different values of the variance σ, until the algorithm produced a fit with a co-variance value higher than 99.5%.

The experimental particle concentration during the irradiation time is evaluated using the Lambert and Beer’s Law:


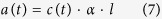


where *a* is the optical absorbance of the colloidal solution, *c* the particle concentration, *α* the molar extinction cross section (tabulated for each wavelength in ref. 11) and *l* (fixed at 10 mm) the optical path length in our experimental setup. We evaluate the particle concentration of the only primary particles produced by the Laser Ablation of the surface, at the wavelength where we observe the plasmonic peak in the extinction cross section of each analyzed spectrum. The optical absorption *a(t),* for a fixed time and wavelength of the plasmonic peak, is obtained directly from the experimental data and is evaluated as:


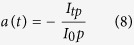


where I_tp_ and I_0p_ are, respectively, the transmitted and initial source intensity at a given wavelength of the plasmonic peak.

Finally, the surface temperature during the laser irradiation is estimated using the results of a thermal model for ns pulsed lasers as described in ref. 12, based on the kinetics of the thermal evaporation and of the condensed bodies. We assume that the entire desorbed material aggregates in NPs, so that the thickness of the ablated material layer at the time *t, h(t)*, can be evaluated from the NP concentration *c* and from the volumetric and geometric parameters of our solution (the erosion level was confirmed by morphological analysis of the irradiated targets before and after the particle production). The surface temperature as function of the irradiation time can be estimated from the formula:


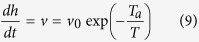



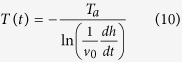


where *v* is the evaporation rate, *T* the surface temperature and *v*_*0*_ and *T*_*a*_ are material’s parameters obtained from reference data^13^.

## Results and Discussion

**Figure 2:**
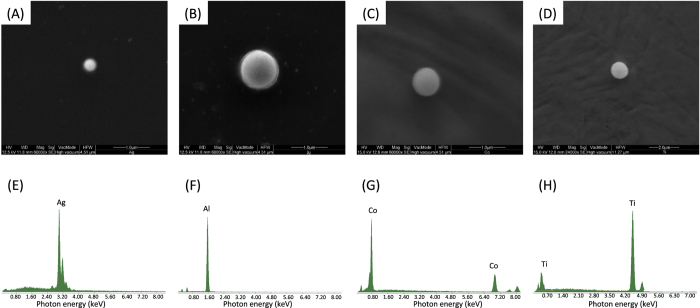
Morphological and chemical analysis of a single particle. (**A**–**D**) SEM images and EDX (**E**–**H**) spectra for Ag, Al, Co, and Ti NPs respectively.

SEM analysis conducted on a droplet of NPs deposed onto a silicon dioxide surface (Fig. 2A–D) confirms that the particles in the solution are spherical. EDX spectra for each NP, taken under SEM conditions (Fig. 2E–H), show only the spectral bands characteristic for the respective metals, without any signs of oxygen or external impurities, indicating the formation of pure metal nanoparticles without the presence of oxides or radicals due to the crack of solvent molecules during the irradiation process.

**Figure 3:**
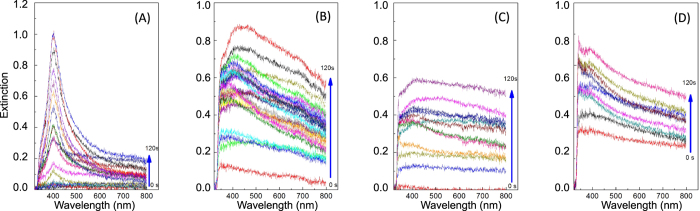
Extinction cross spectra taken at regular time intervals of 2 s during the LASiS process for the production of Ag (**A**), Al (**B**), Co (**C**), and Ti (**D**) NPs. Only a subset of the spectra is shown in the Figure.

**Figure 4:**
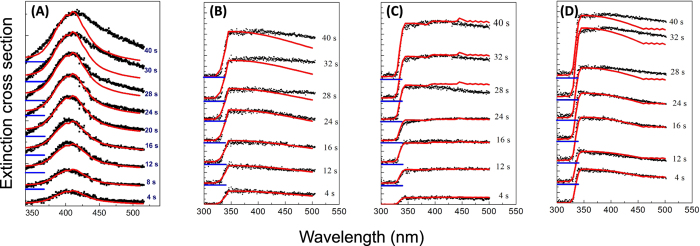
Theoretical fit for all synthesized particles. Fit of the Mie extinction section with a set of representative extinction cross sections of Ag NPs (**A**), Al NPs (**B**), Co (NPs), and Ti NPs (**D**) taken at different irradiation times. The blue lines indicate the zero for each Extinction cross section.

A selection of the extinction cross-sections, taken during the laser irradiation and at regular intervals of 2 s, is shown in Fig. 3 for all the considered nanoparticle types (Ag in A, Al in B, Co in C, and Ti in D). It is clear that the position of the plasmonic oscillation stays constant for each material during the entire laser irradiation process while the extinction shape is regular only in the first 30–35 s of the irradiation time and changes for longer irradiation times. In particular, we can observe that for longer irradiation times the spectra become broader with a widening for wavelengths greater than 500 nm, broadening which gradually increases with irradiation time. In the following, we analyze in detail the fits performed for all the synthesized nanoparticles (Fig. 4A for Ag NPs, 4B for Al NPs, 4 C for Co NPs, and 4D for Ti NPs).

The fit with the calculated extinction curve, shown in Fig. 4A for the Ag NPs, indicate a good covariance for irradiation times below 28 s (correlation greater than 99.5% for spectra taken up to 26 s of irradiation) and the impossibility to fit the green-red spectral region after this irradiation time. This behavior clearly indicates the formation, after the first 26–30 s of irradiation, of NPs with non-spherical shapes and with dimensions becoming gradually larger, and the starting of an aggregation process occurring between the primary NPs produced by LA irradiation (with a passage from region A to B and OR). The results of the fit parameters indicate the formation of primary particles with a radius below 10 nm for all the investigated metals, and a behavior following a LogNorm distribution (see values of the radius and the LogNorm variance indicated in Table 1).

**Figure 5:**
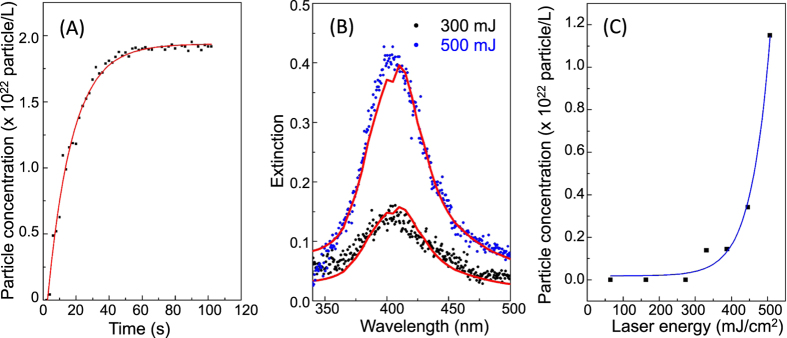
Particle concentration. (**A**) Ag NP concentration as function of the irradiation time; (**B**) Fit of two extinction spectra taken after 15 s for a laser fluence of 300 mJ/cm^2^ and 500 mJ/cm^2^; (**C**) Ag NP concentration as function of the laser fluence.

The analysis of the evolution in time of the only primary particle concentration, evaluated using the first plasmonic peak for each spectrum, indicates that the concentration of smaller particles linearly increases until 20–30 s and then reaches a saturation regime (see Fig. 5A for the Ag NPs as example for all NPs species), while the aggregation of particles becomes the main phenomenon in the plume.

Changes in the laser fluence (performed in the range from 50 mJ/cm^2^ to 500 mJ/cm^2^) do not influence the nucleation/aggregation behavior of the nanoparticles and the dimensions of the primary particles, but modify the value of the particle concentration. Spectra taken at the same irradiation time with different laser fluences (Fig. 5B showing Ag NPs after 15 s of irradiation with a laser having a fluence of 300 and 500 mJ/cm^2^ respectively) can be fitted with the same extinction curve and as such confirm the same parameters for the radius and the LogNorm variance of the primary particle. Nevertheless, for different fluences we observe quite different intensities of the plasmonic peak, indicating an unlike particle concentration in the produced solution. The particle concentration (evaluated after 15 s of the irradiation) as function of the laser fluence is shown in Fig. 5C. We do not observe a particle formation for a fluence below 300 mJ/cm^2^ which points to a threshold behavior in the Laser Ablation process (threshold value ϕ_t_). For a fluence ϕ > ϕ_t_ we observe an exponential increase in the particle concentration (and therefore also in the amount of the ablated material) typical for materials that strongly absorb the laser radiation (such as metals)^14^.

The threshold behavior of the Laser Ablation and the nucleation/aggregation growth can be explained in terms of thermal evaporation of atoms from the surface and their successive aggregation in a plasma plume. Theoretical results related to the thermal mode (see ref. 12) indicate that the temperature in the first ns of irradiation (of the order of the laser pulse duration) is in a range between 2000–3000 K, which is the typical evaporation temperature of metals (2000 K for silver). For longer irradiation times, the surface temperature decreases below 1000 K, as confirmed by our experimental data. The calculation of the surface temperature using equations 9 and 10 yields to a value between 360–380 K. Given the above-mentioned facts, it is likely that the metal atoms evaporate from the bulk surface during the irradiation with the laser pulse before the NPs nucleate then nucleate in a plasma plume forming primary NPs. When the primary particle concentration becomes high, an aggregation mechanism begins to setup between the primary particles and the atoms evaporated from the surface; as a result, large amorphous particles appear in the solution. The aggregation occurs randomly and with increasing irradiation time leads to the formation of greater cluster. In addition, the extinction spectrum becomes very broad, the color of the solution darkens, and the Mie theory does not fit anymore with the experimental data (see Fig. S1 in the Supporting Materials). The absence of secondary plasmonic peaks indicates that the produced aggregates assume an amorphous shape with a complete absence of any spherical configuration having a radius greater than the primary particles.

**Figure 6:**
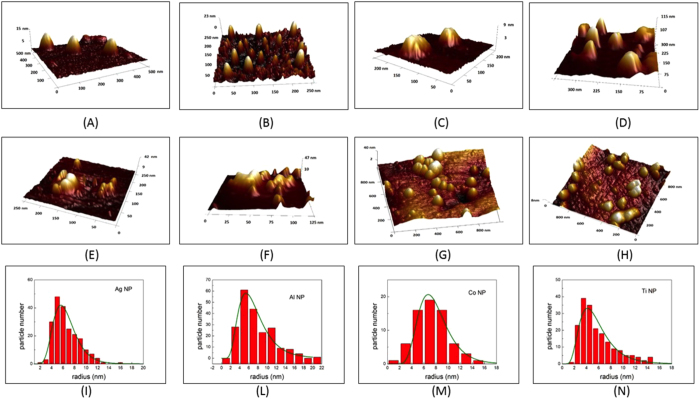
Statistical analysis from AFM. AFM 3D images of NPs obtained after an irradiation time of 15 s (Ag in (**A**), Al in (**B**), Co in (**C**), and Ti in (**D**)) and of 60 s (Ag in (**E**), Al in (**F**), Co in (**G**), and Ti in (**H**)); Histograms of the NP radius distribution (Ag in (**I**), Al in (**L**), Co in (**M**), and Ti in (**N**)).

**Figure 7:**
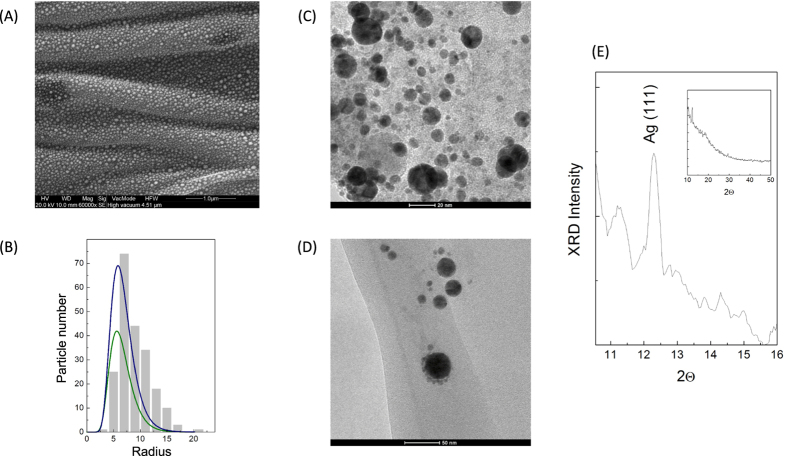
Statistical analysis from SEM. (**A**) SEM images of Ag NPs obtained after an irradiation time of 15 s and (**B**) histogram of the NP radius distribution, the two curves in (**B**) represent the LogNorm distributions obtained by the AFM (green) and SEM (blue) for comparison; (**C**,**D**) TEM images of Ag NPs collected after 30 s of irradiation and (**E**) XRD analysis of the Ag NPs indicating the crystalline nature of the particles.

Morphological analysis, conducted by SEM, TEM and AFM, of a droplet of the solution taken at two different irradiation times, one before and one after the start of the aggregation (15 s and 60 s) confirm the results of the absorption analysis: For short irradiation times we observe in the AFM and SEM images (see the AFM images in Fig. 6A,B,C and D for respectively Ag, Al, Co and Ti; and the SEM image for Ag NPs in 7A) for SEM) the formation of nanoparticles with different dimensions. The radius distributions (Figs 6I to N and 7B), evaluated with the Bearing and statistical analysis (using the AFM and SEM respectively), as described in the Methods section, indicate that the NP radius are distributed as a LogNorm function with parameters of mean value and variance corresponding to those obtained from the Mie analysis (see comparison in tab. 1 and in Fig. 7B for the LogNorm distribution obtained from AFM and SEM). For irradiation times greater than 30 s, morphological analysis (Fig. 6E,F,G,H for Ag, Al, Co, and Ti respectively for AFM and 7 C and 7D for TEM) shows the presence of large amorphous aggregates with NP dimensions varying from a few tens of nm to hundreds of nm, without any sign of a regular distribution. Moreover, XRD analysis reveals the formation of crystalline particles in the first 30 s (Fig. 7E show the analysis for Silver particles (Ag (111)), as example for all). In addition, the produced particles are pure (i.e. not showing any presence of oxygen) and their dimension, shape, and concentration can exactly be controlled monitoring the optical absorption during the measurements.

## Conclusions

We have investigated the aggregation of metallic nanoparticles in a colloidal solution during the Laser Ablation growth process with the aim of identifying the exact nucleation and aggregation phases that occur during the laser irradiation process. This, in order to allow for a better control of the growth process and consequently the NP parameter. We demonstrate that the laser irradiation produces primary NPs with radius less than 10 nm in the first 25–28 s of the laser irradiation before an aggregation process starts that forms amorphous aggregates whose dimensions increase with irradiation time. An estimation of the surface temperature from the experimental data, and the comparison with the results of a Thermal Model for the Laser Ablation, indicate that the nucleation mechanism is based on the evaporation of atoms from the sample surface during the first ms of the irradiation and a nucleation of these atoms into a plasma plume. Dimension, shape, and concentration of nanoparticles in the colloidal solutions can be precisely monitored using optical absorption performed during the irradiation process and fitting the extinction spectra with the theoretical prevision of the Mie theory. However, strong effort needs still to be made in order to understand the exact mechanism of the nucleation of the primary particles on a shorter temporal scales, i.e. on a ns scale.

## Additional Information

**How to cite this article**: Barberio, M. and Antici, P. *In situ* study of nucleation and aggregation phases for nanoparticles grown by laser-driven methods. *Sci. Rep.* **7**, 41372; doi: 10.1038/srep41372 (2017).

**Publisher's note:** Springer Nature remains neutral with regard to jurisdictional claims in published maps and institutional affiliations.

## Supplementary Material

Supplementary Material

## Figures and Tables

**Table 1:** NP mean radius and variance evaluated with a fit of the extinction cross sections and using morphological analysis.

Nanoparticle	Bearing Analysis from AFM analysis		Particle radius from SEM analysis		Mie Fit	
	Radius (nm)	σ (nm)	Radius (nm)	σ (nm)	Radius (nm)	σ (nm)
Ag	6.7	2.1	7.1	2.2	6.5	2.5
Al	7.4	3.6	7.8	2.2	7.8	2.2
Co	7.9	2.7	8.2	2.1	8.0	2.9
Ti	5.8	2.9	5.2	2.1	6.5	3.0
