# Supplementary material for: In situ study of nucleation and aggregation phases for nanoparticles grown by laser-driven methods
Source: Sci Rep. 2017 Feb 14;7:41372. doi: 10.1038/srep41372 (PMC5307318; doi:10.1038/srep41372)
Supplement: Supplementary Material [file srep41372-s1.pdf]

Supporting Materials

In situ study of nucleation and aggregation phases for nanoparticles grown by laser-driven methods

M. Barberio[1], P. Antici[1]

[1]*INRS-EMT, 1650 Boul. Lionel Boulet, Varennes, J3X1S2, Canada*

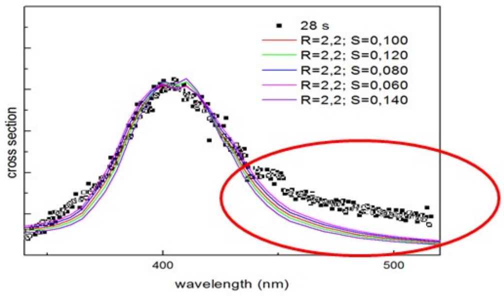

Supporting 1: Five different tentative fits using a larger variance in the convoluted LogNorm function. The proposed fits do not converge in the region of 500 nm.
